# Supplementary material for: Identification of photoperiod-induced specific miRNAs in the adrenal glands of Sunite sheep (Ovis aries)
Source: Front Vet Sci. 2022 Jul 22;9:888207. doi: 10.3389/fvets.2022.888207 (PMC9354845; doi:10.3389/fvets.2022.888207)
Supplement: Supplementary file 1 [file Data_Sheet_1.ZIP › Supplementary file/miRDeep2 file.zip!/miRDeep2 file/Novel_Structure/LP42A2/Novel_244.pdf]

Provisional ID	:	NC_019472.2_25375
Score total	:	2
Score for star read(s)	:	-1.3
Score for read counts	:	0
Score for mfe	:	1.7
Score for randfold	:	1.6
Score for cons. seed	:	
Total read count	:	484
Mature read count	:	482
Loop read count	:	0
Star read count	:	2

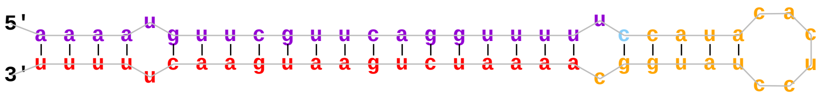

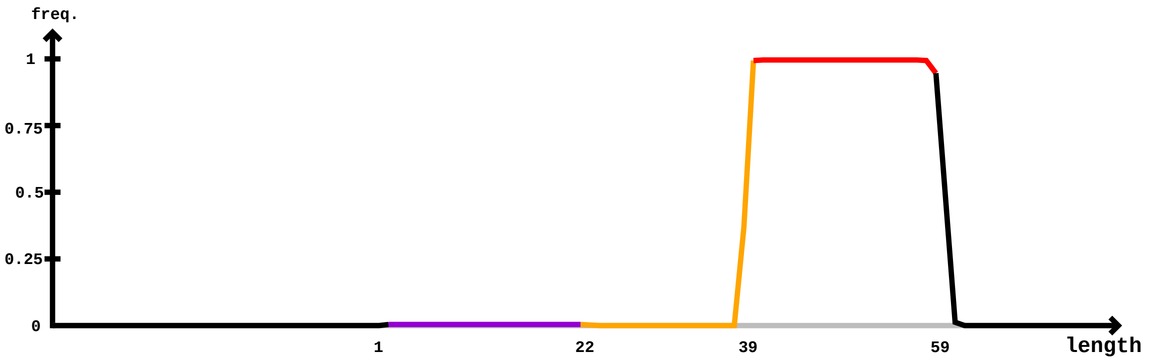

Star Mature

```
5'- ugaagcacaaaaagcuuauguauuggguuggccaaaauguucguucagguuuuuccauacacuccuauggcaaaaucugaaugaacuuuuugcccauuugaacucaauc   -3'    obs
    ugaagcacaaaaagcuuauguauuggguuggccaaaauguucguucagguuuuucauacacuccuauggcaaaaucugaaugaacuuuuugcccauuugaacucaauc          exp
    ..((((.......))))....(((((((((.(((((.((((((((((((((.(((((......))))).))))))))))))))).))))).))).....)))))))).   reads    mm    sample
    .................................aaaaAguucguucagguuuuu.......................................................   2        1     seq
    .....................................................................caaaaucugaaugaacuuu.....................   1        0     seq
    .....................................................................Aaaaaucugaaugaacuuuuu...................   111      1     seq
    .....................................................................caaaaucugaaugaacuuuuu...................   3        0     seq
    .....................................................................caaaaucugaaugaacuuuuug..................   3        0     seq
    .....................................................................Aaaaaucugaaugaacuuuuug..................   59       1     seq
    .....................................................................Gaaaaucugaaugaacuuuuug..................   1        1     seq
    ......................................................................aaaaucugaaugaacuuuu....................   23       0     seq
    ......................................................................aaaaucugaaugaacuuuuG...................   2        1     seq
    ......................................................................aaaaucugaaugaacuuuCu...................   2        1     seq
    ......................................................................aaaaucugaaCgaacuuuuu...................   6        1     seq
    ......................................................................aaaaucugaaugaacuuuuu...................   258      0     seq
    ......................................................................aaaaucAgaaugaacuuuuug..................   1        1     seq
    ......................................................................aaaaucugaaugaGcuuuuug..................   1        1     seq
    ......................................................................aaaaucugaaugaacuuuuGg..................   1        1     seq
    ......................................................................aaaaucugaaugaacuuuuuC..................   3        1     seq
    ......................................................................aaaaucugaaugaacuuuuugc.................   6        0     seq
    .......................................................................aaaucugaaugaacuuuuu...................   1        0     seq
```
